# First experience with a vendor-neutral three-dimensional mapping system for cardiac magnetic resonance-guided electrophysiological procedures: a case report

**DOI:** 10.1093/ehjcr/ytae271

**Published:** 2024-06-04

**Authors:** Giulia De Zan, Marjolein de Jongh, Vjeran Karloci, Marco Guglielmo, Ivo van der Bilt

**Affiliations:** Department of Thoracic, Heart and Vascular Diseases, Maggiore della Carità Hospital, Novara, Italy; Department of Cardiology, Division of Heart and Lungs, Utrecht University, Utrecht University Medical Center, Heidelberglaan 100, 3584 CX, Utrecht, The Netherlands; Department of Cardiology, Haga Teaching Hospital, Els Borst-Eilersplein 275, 2545 AA, The Hague, The Netherlands; Department of Interventional CMR, Imricor Medical Systems, Burnsville, MN, USA; Department of Cardiology, Division of Heart and Lungs, Utrecht University, Utrecht University Medical Center, Heidelberglaan 100, 3584 CX, Utrecht, The Netherlands; Department of Cardiology, Haga Teaching Hospital, Els Borst-Eilersplein 275, 2545 AA, The Hague, The Netherlands; Department of Cardiology, Division of Heart and Lungs, Utrecht University, Utrecht University Medical Center, Heidelberglaan 100, 3584 CX, Utrecht, The Netherlands; Department of Cardiology, Haga Teaching Hospital, Els Borst-Eilersplein 275, 2545 AA, The Hague, The Netherlands

**Keywords:** Mapping system, Interventional cardiac magnetic resonance, Catheter ablation, Electrophysiology, Case report

## Abstract

**Background:**

Fluoroscopy-guided catheter ablation has become the gold standard for treatment of cardiac arrhythmias. High resolution electro-anatomical mapping systems have become fundamental to perform these procedures. Recently, interventional cardiac magnetic resonance (iCMR) has been proposed as an alternative for fluoroscopy to guide atrial flutter ablations. The clinical experience with iCMR and dedicated three-dimensional mapping systems is growing. NorthStar is currently the first available vendor-neutral mapping system.

**Case summary:**

We performed a real-time CMR-guided cavotricuspid isthmus (CTI) catheter ablation (CA) on a 69-year-old man using a novel mapping system (NorthStar Mapping System, Imricor Medical Systems, MN, USA). Starting from the CMR imaging, a pre-rendered segmentation model was loaded on NorthStar and used to guide the catheters, display voltage and activation maps, show mapping and ablation points. NorthStar can also take full control of the CMR scanner (i.e. start/stop sequences for anatomical information, tissue characterization, and catheter visualization) and communicate with the recorder/stimulator system (Advantage-MR EP, Imricor Medical Systems, MN, USA). With comparable procedural time to standard fluoroscopy-guided CA, CTI bidirectional block was achieved, without any complication.

**Discussion:**

Using the NorthStar Mapping System, we managed to achieve a successful CMR-guided CTI ablation without any complication. Its further use should be explored, especially in more complex arrhythmias where a substrate-guided ablation is critical, as it could significantly improve results in terms of arrhythmia recurrence.

**Learning points**

- To describe the functioning and usefulness of a new mapping system in the interventional cardiac magnetic resonance (CMR) environment for electrophysiological procedures.
- To spread the knowledge around real-time CMR-guided ablations and show the potentials of a vendor-neutral mapping system.

## Introduction

Cardiac arrhythmias represent a major health burden, as they are associated with significant co-morbidity (such as heart failure, stroke, and decreased quality of life) and increased mortality. Current guidelines suggest fluoroscopy-guided catheter ablation (CA) as a valuable option for the treatment of arrhythmias, being the first choice over antiarrhythmic medications in atrial flutter (AFL) and in selected case of atrial fibrillation (AF).^1–3^ Nonetheless, arrhythmia recurrence rate following an initially successful CA is still high, despite technological developed equipment, including high resolution mapping systems.^4,5^ This is due to either technical failure in forming transmural lesions, typical of AF recurrence, or incorrect selection of ablation targets, which is essential in VT ablation.^6^ With its ability of tissue characterization, cardiac magnetic resonance (CMR) has recently emerged as a promising technique to guide CA using a substrate-based, anatomical approach.^7^ First clinical experiences were done in the field of cavotricuspid isthmus (CTI) ablation and showed similar safety and efficacy compared to conventional fluoroscopy-guided ablation.^7–9^ However, a vendor-neutral mapping software for real-time CMR-guided CA has been lacking. Recently, the NorthStar Mapping System (Imricor Medical Systems, MN, USA) has been developed to fill this gap. The present paper is meant to describe the first experience using the NorthStar Mapping System in a clinical setting.

## Summary figure

**Figure:**
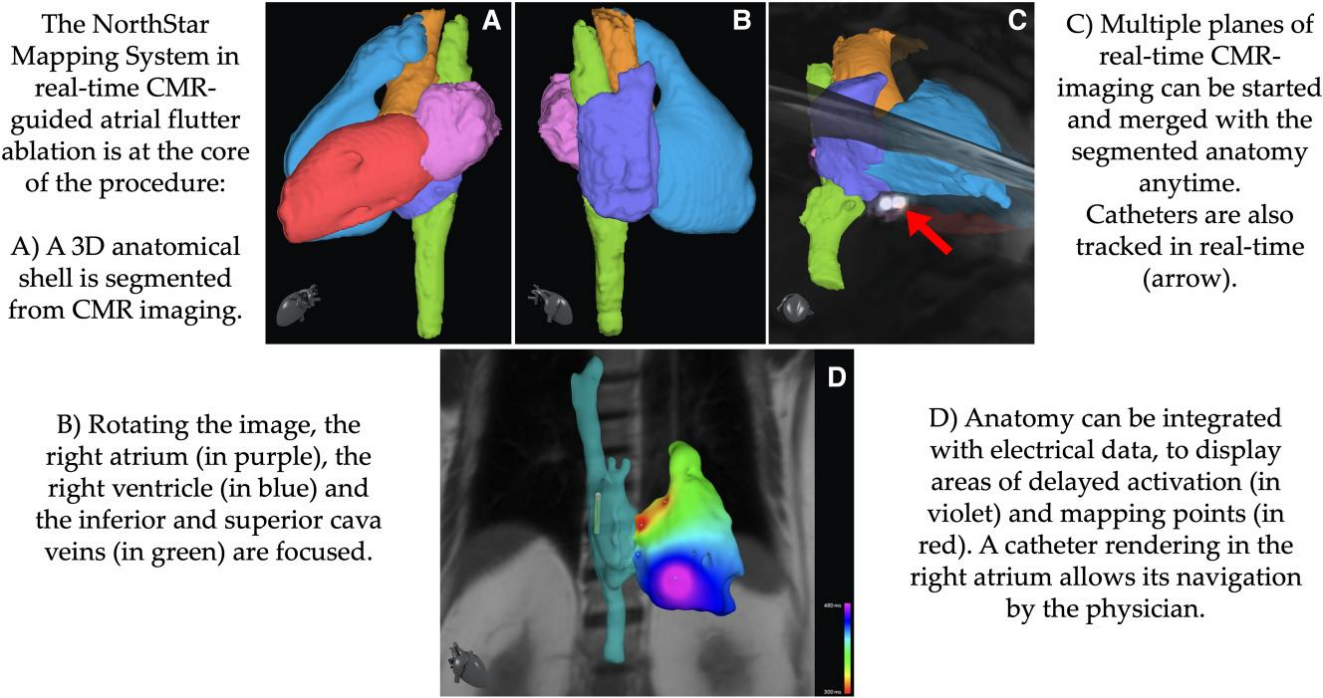


## Case presentation

A 69-year-old man was referred to our outpatient clinic with daily episodes of palpitations and an electrocardiographic diagnosis of a typical counterclockwise AFL. The patient had no major comorbidities and no cardiovascular risk factors apart from overweight (body mass index = 28 kg/m^2^). Physical examination and lab results were unremarkable. A transthoracic echocardiogram showed a structurally normal heart with only a slightly dilated LA. A real-time CMR-guided CTI CA using the NorthStar Mapping System was offered to the patient.

**Figure 1:**
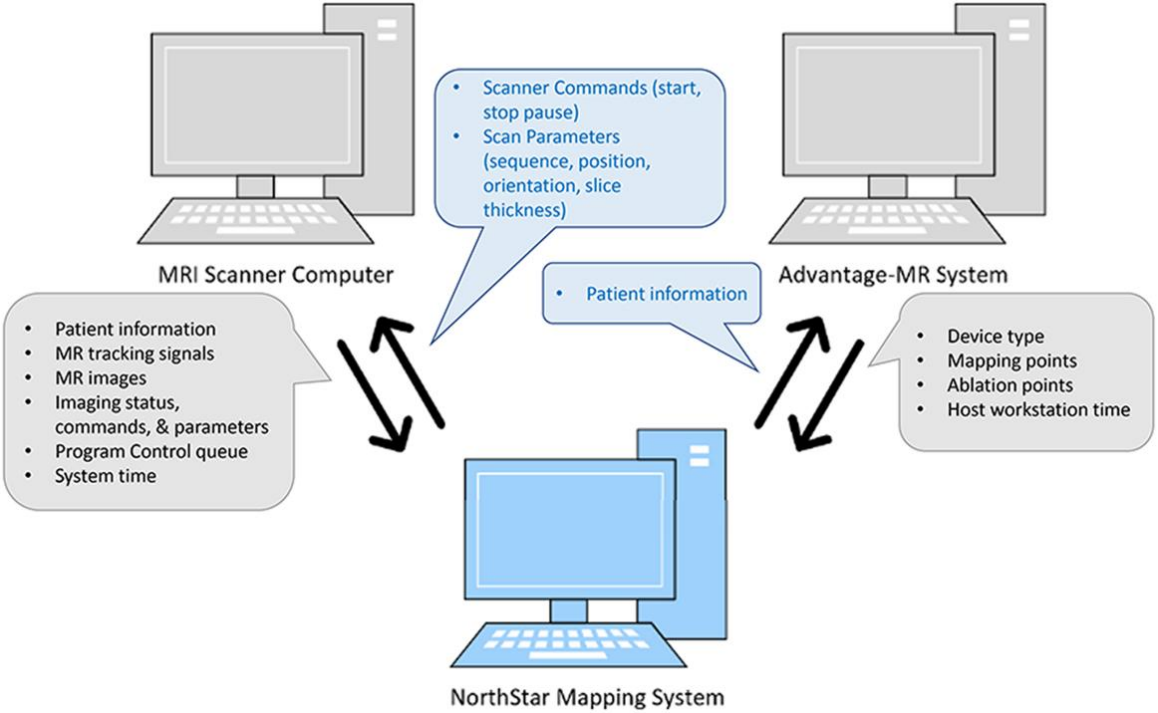
The efficiency of the NorthStar Mapping System in guiding the ablation depends strictly on the interconnection between the software, the scanner computer and the Advantage-MR System. In the figure, it is shown which data are sent and shared between each device.

The procedure started with the acquisition of a full volume anatomy of the heart and surrounding structures, using ECG-triggered respiratory-navigated 3D steady state free precession sequences in a conventional 1.5 Tesla MRI unit (MAGNETOM Aera, Siemens Healthcare, Erlangen, Germany). From this CMR images, a 3D shell of the cardiovascular anatomy was obtained using the segmentation software ADAS 3D (ADAS 3D Medical, Barcelona, Spain). The shell was then exported and uploaded in the NorthStar Mapping System, where it was displayed according to the spatial coordinates of the MRI scanner. NorthStar was connected to both the CMR computer and the Advantage-MR EP Recorder/Stimulator System (Imricor Medical Systems, MN, USA) via a dedicated Ethernet socket interface (*Figure 1*). The resulting communication between these devices allowed:

- pre-selected sequences to be initiated and stopped by NorthStar;
- real-time display of CMR images on NorthStar via an automatic upload from the CMR scanner; and
- combination and display in a 3D environment of CMR imaging, imported 3D shells and substrate maps, catheter location, mapping and ablation points, and voltage and activation maps.

Subsequently, two MRI-conditional catheters (Vision-MR Catheters, Imricor Medical Systems, Burnsville, MN) were inserted through a femoral venous access. The Vision-MR is a bipolar catheter, with a 1.3 mm spacing between the two electrodes, and it has a 115 cm usable length, 9 French diameter and 32 mm curve diameter. The catheters were real-time displayed thanks to two micro-coils on each catheter tip. Active catheter imaging (ACI) sequences display the receive coil as a bright spot within the CMR images. Conversely, active catheter tracking (ACT) is no imaging technique, but a tracking technique. Applying three subsequent orthogonal magnetic field gradients, the frequency of the spins immediately next to the micro-coils changes. Since the frequency of the spins is dependent on their relative position to the magnetic field, a final 3D position of the micro-coils is reconstructed. NorthStar then translates this position in a rendering for catheter manipulation. Moreover, ACT allows scan sequence manipulation based on the catheter position and orientation, and it is faster than ACI, allowing the tracking of the catheter 20 times per second. Because of a 10° flip angle, the scanner noise is greatly reduced.

**Figure 2:**
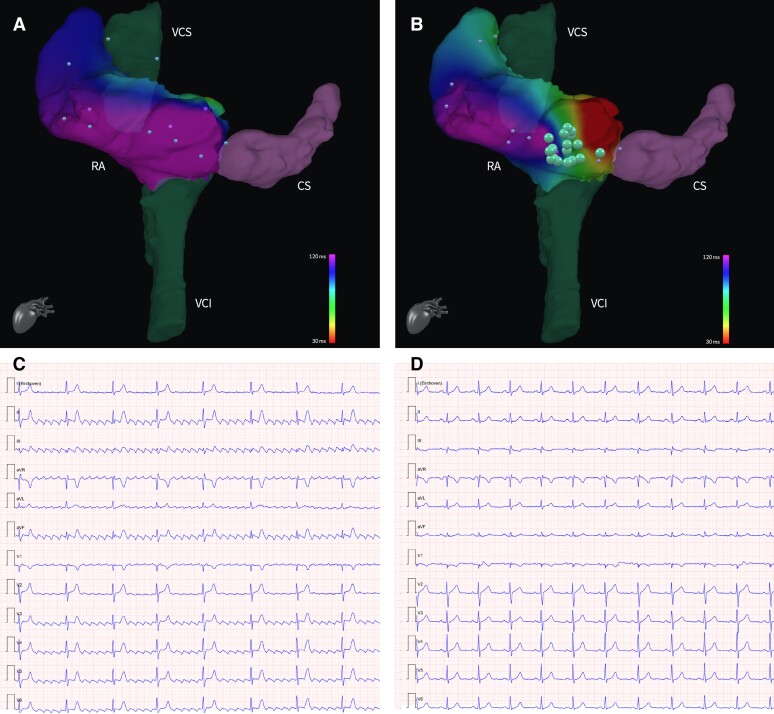
Data of mapping points (little dots) derived from the Advantage-MR EP Recorder/Stimulator System can be used to create activation maps to superimpose on the 3D anatomical shell. Panel (*A*) shows the activation map of the RA of our patient at the beginning of the procedure, corresponding to a typical counterclockwise atrial flutter (*C*). After the ablation, the new activation map reflects the achievement of a bidirectional bock at the level of the isthmus (*B*), with achievement of sinus rhythm (*D*). Ablation points (big dots) can interactively show information as power, duration, impedance drop, and temperature associated to the RF delivery. 3D, three-dimensional; CS, coronary sinus; IVC, inferior vena cava; RA, right atrium; RF, radiofrequency; SVC, superior vena cava.

**Figure 3:**
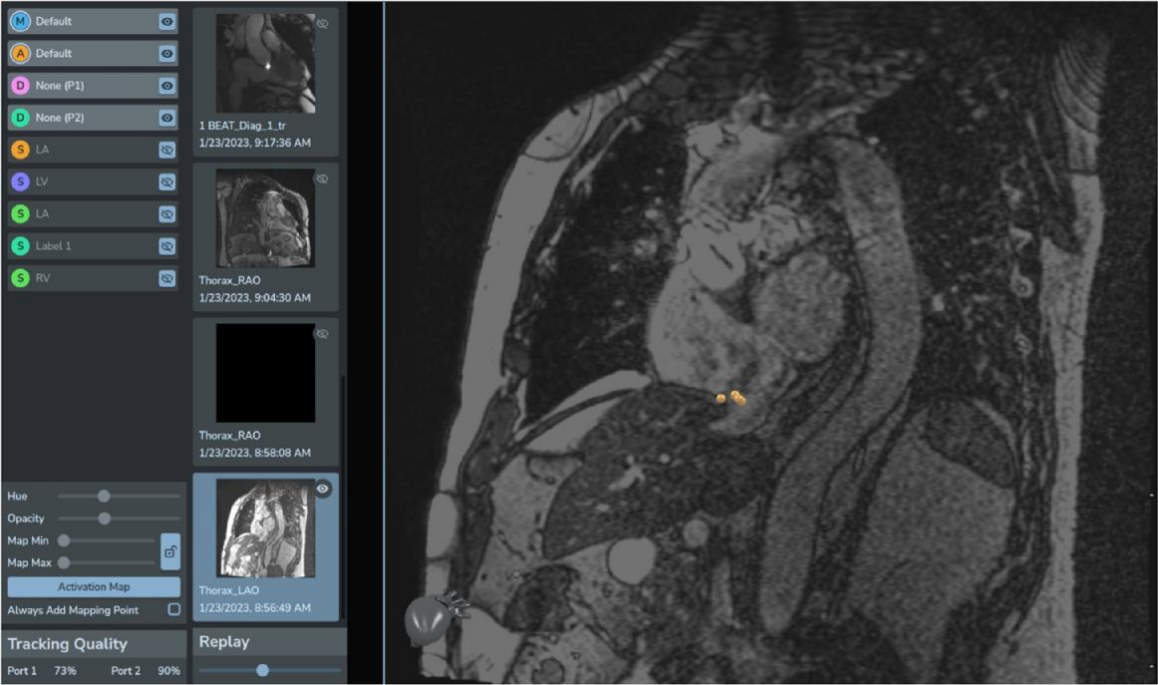
Example of the display of ablation points directly on real-time MRI images without the superimposed 3D shell. Three different ablation points are correctly localized on the CTI, confirming the good orientation of the 3D shell and thus its reliability in guiding the procedure. 3D, three-dimensional; CTI, cavotricuspid isthmus.

**Figure 4:**
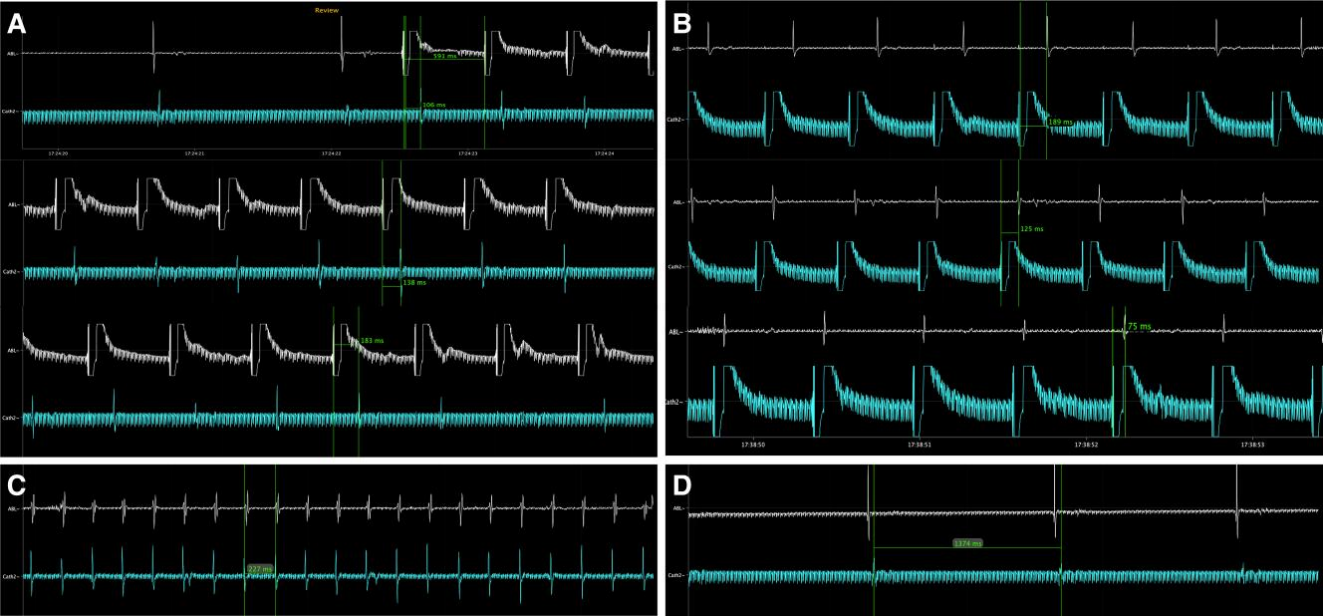
Bidirectional block was demonstrated by pacing on either side of the ablation line. In Panel (*A*), the ablation catheter was used to perform pacing with a standard cycle length of 600 ms, while moving it from the medial end of the CTI, to the superior portion of the RA and eventually to the lateral end of the CTI. The catheter in the CS shows a progressive increase in its activation times, suggesting CTI block. The same protocol was performed via pacing from the CS catheter and measure of the activation times of the ablation catheter (*B*). Panels (*C*) and (*D*) display respectively the atrium electrograms during atrial flutter and after the ablation with achievement of SR. CS, coronary sinus; CTI, cavotricuspid rhythm; RA, right atrium; SR, sinus rhythm.

Once in the right atrium, insertion of one of the catheters in the coronary sinus using ACT was achieved fast and smoothly. At this point, CTI ablation was performed using ACT to guide the catheter towards the CTI and ACI to confirm its position before releasing radiofrequency. Switching from ACT to ACI before delivering of radiofrequency is advised, as ACT visualizes the catheter position in the coordinates of the CMR scanner and not in relation to the imported shell. Ablation was performed with radiofrequency deliveries at 45 W for 30 s each, with interruption of the arrhythmia and achievement of SR (*Figure 2*). NorthStar can display ablation points also on real-time MRI imaging (*Figure 3*). Each ablation point provided information such as duration, power, temperature, and impedance drop. Isthmus block was confirmed by differential pacing, i.e. pacing on either side of the ablation line. The ablation catheter, while performing pacing, would be moved from the medial end to the lateral end of the ablation line, and a progressive lengthening of activation time of the catheter in the coronary sinus was demonstrated (*Figure 4*). The total duration of the procedure (from the first MRI sequence to achievement of SR) was 90 min. The times required for coronary sinus cannulation and ablation (from the first to the last radiofrequency delivery) were respectively 1 and 50 min. No complication occurred. At a 7-month follow-up, the patient is free of arrhythmia recurrence.

## Discussion

To the best of our knowledge, this was the first time the NorthStar Mapping System was ever used to perform a procedure in a human patient. Its application in a CMR-guided CTI CA turned out to be feasible, safe, and successful. The software is specifically designed to integrate the information provided by the MRI, such as cardiovascular anatomy and tissue characterization, with the information of the electrophysiological equipment during iCMR interventions. Therefore, it is meant to play a central role in the ecosystem of iCMR. The NorthStar Mapping System provides visualization of a 3D dataset of cardiac anatomy derived from and fused with real-time CMR imaging, using the full variation of available CMR contrasts, fast planning of scan geometry in 3D, and catheter tip rendering. Moreover, NorthStar can remotely perform validated CMR scan protocols, including specific protocols for CMR-conditional catheter localization, thanks to miniature receive coils. All these features, while being not a novelty *per se*, make NorthStar a versatile tool for iCMR-guided procedures. Differently from iSuite (Philips, Amsterdam, The Netherlands), the other available yet investigational product for CMR-guided procedures, NorthStar is vendor neutral and can potentially connect to and work with the CMR scanner irrespective of its brand. This is of utmost importance and could make CMR-guided procedures more approachable and attractive by centres that do not perform them yet.^10^

Moreover, we recognize that CMR-guided CA could be particularly useful in the treatment of complex arrhythmias such as VTs. However, treatment of cardiac arrhythmias with a simpler electro-anatomical substrate, such as AFL, seems to be the necessary and most logical first step to gain experience with this novel technique and software. In fact, the features of the NorthStar Mapping System and our early experience in CTI ablation support its further use in other cardiac arrhythmias, such as VTs, to express the full potential of iCMR, guiding physicians in a substrate-targeted ablation and, therefore, improving patient care.

## Data Availability

The data underlying this article are available in the article.

## Lead author biography

Dr Giulia De Zan graduated from Bologna University in Italy in 2017 and trained in Cardiology at Eastern Piedmont University (Italy) and University Medical Center Utrecht (The Netherlands). Her research activity focuses on cardiac magnetic resonance imaging, with a special interest in interventional MRI.
